# Identification of Novel ROR2 Gene Mutations in Indian Children with Robinow Syndrome

**DOI:** 10.4274/jcrpe.1233

**Published:** 2014-06-05

**Authors:** Parag M Tamhankar, Lakshmi Vasudevan, Shweta Kondurkar, Yashaswini K, Sunil Kumar Agarwalla, Mohandas Nair, Ramkumar TV, Nitin Chaubal, Vasundhara Sridhar Chennuri

**Affiliations:** 1 Genetic Research Center, NIRRH, Mumbai, India; 2 The Maharaja Krishna Chandra Gajapati Medical College & Hospital, Department of Pediatrics, Berhampur, India; 3 Government Medical College, Department of Pediatrics, Kozhikode, Kerala, India; 4 Great Eastern Medical School, Department of Pediatrics, Srikakulam, India; 5 Thane Ultrasound Center, Thane, India; 6 Employees State Insurance Corporation Medical College, Department of Pediatrics, Thane, İndia

**Keywords:** Short limbed dwarfism, mesomelia, facial dysmorphism, ambiguous genitalia, mutation, ROR2 gene related disorders, WNT5A gene

## Abstract

**Ob­jec­ti­ve:** Robinow syndrome (RS) is an extremely rare genetic disorder characterized by short-limbed dwarfism, defects in vertebral segmentation and abnormalities in the head, face and external genitalia. Mutations in the ROR2 gene cause autosomal recessive RS (RRS) whereas mutations in WNT5A are responsible for the autosomal dominant (AD) form of RS. In AD Robinow patients, oral manifestations are more prominent, while hemivertebrae and scoliosis rarely occur and facial abnormalities tend to be milder.

**Methods:** Three unrelated patients from different parts of India were studied. These patients were diagnosed as RRS due to presence of characteristic fetal facies, mesomelia, short stature, micropenis, hemivertebrae and rib abnormalities. One of the patients had fetal facies and micropenis but unusually mild skeletal features. This patient’s mother had mild affection in the form of short stature and prominent eyes. Testosterone response to human chorionic gonadotropin was investigated in two patients and were normal. The exons and exon-intron boundaries of the ROR2 gene were sequenced for all probands. Bioinformatics analysis was done for putative variants using SIFT, PolyPhen2 and Mutation Taster.

**Results:** Patients 1, 2 and 3 were homozygous for c.G545A or p.C182Y in exon 5, c.227G>A or p.G76D in exon 3 and c.668G>A or p.C223Y in exon 6 respectively. Prenatal diagnosis could be performed in an ongoing pregnancy in one family and the fetus was confirmed to be unaffected.

**Conclusion: **ROR2 mutations were documented for the first time in the Indian population. Knowledge of the molecular basis of the disorder served to provide accurate counseling and prenatal diagnosis to the families.

## INTRODUCTION

Robinow syndrome (RS) is an extremely rare genetic disorder characterized by short-limbed dwarfism, defects in vertebral segmentation and abnormalities in the head, face and external genitalia. The disorder was first described in 1969 by human geneticist Robinow et al (1). Two types of RS exist which are distinguished by the severity of their signs and symptoms and by their patterns of inheritance. Mutations in the ROR2 gene cause the more common autosomal recessive RS (RRS) (2). The gene encoding receptor orphan receptor tyrosine kinase 2 (ROR2) is located on chromosome 9q22 (OMIM 602337) and homozygous or compound heterozygous loss of function mutations in this gene are responsible for RRS (3,4). Mutations in WNT5A, locus 3p14.3 (OMIM 164975) are responsible for the autosomal dominant (AD) form of RS In these patients, oral manifestations are more prominent, hemivertebrae and scoliosis rarely occur and facial abnormalities tend to be milder (4). We report the mutations found in ROR2 gene in three children referred to our institute in the past two years, who had clinical and radiological features suggestive of the recessive form of RS. 

## METHODS

**Patients**

**Patient 1.** A male child aged 21/2 years, an offspring of non-consanguineous parents from Uttar Pradesh was referred for short stature and facial dysmorphism. The referring physician had made a tentative diagnosis of achondroplasia prenatally. The proband was reported to be short at birth.

**Figure 1:**
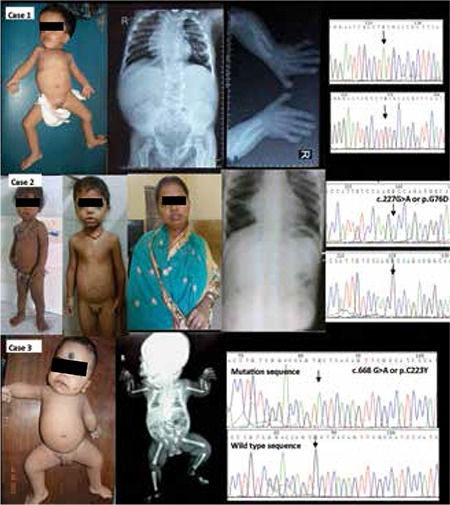
The clinical radiological and molecular findings of the three Indian cases with Robinow syndrome: Case 1 is a male child with acromesomelia, fetal facies, micropenis, vertebral and rib abnormalities. Case 2 presents two brothers with the typical fetal facies and micropenis without vertebral or rib anomalies; and the mother with short stature and prominent eyes. Case 3 shows the child from Kerala with the typical fetal facies, micropenis and forked ribs. Sequence chromatograms of homozygous mutations are shown on the right hand side of the respective cases

At presentation, the child’s motor and mental milestones were appropriate for age. His height was 67.5 cm [-7.1 standard deviation (SD)], upper segment (US)/lower segment (LS) measurements were 40 cm/27.5 cm (a ratio of 1.5:1 indicating short limbs). His head circumference was 47 cm. He had acromesomelia with facial abnormalities such as frontal bossing, prominent eyes, hypertelorism, flat nasal bridge, anteverted nostrils, folded low set ears, tented upper lip, wide mouth, gum hypertrophy suggestive of “fetal facies”. Other features were thoracolumbar scoliosis, small hands, small penis (stretched penile length 1 cm) and retractile testes. X rays showed a large cranium, crowded right sided ribs, absent ribs 3 and 4 on the left side, marked thoracic scoliosis to the right and lumbar scoliosis to the left, thoracic and lumbar hemivertebrae, short radius and ulna. The upper limb mesomelia was more severe than that of the lower limb. Ultrasonography (USG) and echocardiography examinations were normal (Figure 1, case 1).

HCG was administered IM, in a dose of 3000 IU/m2/day for 3 days. Total serum testosterone was measured at baseline (on day 1 before HCG injection) and on day 4 (1st day after 3 injections) using chemiluminescence immunoassay. The basal testosterone 1 was 2.5 ng/dL (normal prepubertal level is less than 10 ng/dL) and the day 4 level was 165 ng/dL (an increase to above 150 ng/dL is accepted as normal). 

The mother of this patient was in a pregnant state and the parents agreed to undergo genetic analysis for antenatal diagnosis. 

**Patient 2.** A 24 months old male child born to third degree consanguineous parents from Odisha, was referred for molecular diagnosis of RS due to clinical features and radiological findings. The proband was antenatally diagnosed by USG to have short limbs at 32 weeks of gestation. He was delivered at term and birth weight was 2150 g. 

At presentation, the patient had a head circumference of 48 cm (50th centile for age), length of 73 cm (-4.8 SD), US/LS ratio (41 cm/32 cm) was 1.28:1. Clinical findings included a fetal facies, frontal bossing, prominent eyes, lateral eversion of lower eyelids, exposure keratitis, depressed nasal bridge, short upturned nose, long philtrum, small cleft in lower lip, micrognathia, gum hypertrophy, acro mesomelia of upper and lower limbs, short stubby fingers and a buried penis (Figure 1, case 2). Radiological findings were bilateral elbow dislocations, multiple hemivertebrae and forked ribs. The echocardiogram was normal except for a bicuspid aortic valve. Serum electrolyte levels, thyroid function tests and abdominal USG findings were normal. The mother of the proband also had mild facial dysmorphism consisting of a depressed nasal bridge, a short upturned nose, mesomelia of the upper limbs, stubby fingers and toes and short stature. The elder sibling of the proband also, with prominent eyes, eversion of the lower lids, depressed nasal bridge, low set small ears, long philtrum, stubby toes and fingers, mesomelia and buried penis, bore the typical signs of RRS. The father had no signs of dysmorphism. 

**Patient 3.** A male child born to third degree consanguineous parents from Kerala was brought for evaluation of dysmorphic features and small phallus. The mother had undergone two first trimester abortions: The first of these was a medical termination of pregnancy following chickenpox and the second, a spontaneous abortion). Antenatal period for this pregnancy was uneventful except for short stature of the fetus, noticed at 32 weeks by USG. The infant was born by a term delivery with a birth weight of 2150 g. At 17 months of age, his height was 59 cm (below the -4 SD), US/LS ratio 1.3:1. The head circumference was 44 cm (-3 SD) but appeared large compared to body measurements. He had mild global developmental delay, short upper limbs, mainly acro mesomelia, dislocation of both elbows, brachydactyly, clinodactyly, coarse facial features, frontal prominence, prominent eyes with lateral eversion of lower eyelids and exposure keratitis, anteverted nose, a large mouth with small clefts in both upper and lower lips, a long philtrum, gum hypertrophy, micrognathia and micropenis. Skeletal survey showed multiple hemivertebrae, short forearm bones with triangular shaped ulna, short first metacarpals and hypoplastic middle and distal phalanges (Figure 1). The echocardiogram showed a congenital bicuspid aortic valve. A beta human chorionic gonadotropin (HCG) test was performed in this patient too. The basal testosterone and dihydrotestosterone were less than 2.5 ng/dl and 13.90 pg/ml in patient 3 and day 4 level was 453 ng/dl and 139 pg/ml respectively. 

**Methodology for Genetic Analysis**

Sequencing of all exons of ROR2 was performed on an automated capillary sequencer as reported before. Sequences were compared with wild type using online BLAST. Variants obtained were tested for pathogenicity using bioinformatics software SIFT (http://sift.jcvi.org/), PolyPhen2 (PPH2) (http://genetics.bwh.harvard.edu/pph2/) and Mutation Taster (MT) (http://mutationtaster.org/MutationTaster/index.html) (5,6,7). The pathogenic mutations thus obtained were confirmed in the parents by sequencing the corresponding exons. Mutations were also ruled out as polymorphisms by sequencing in 100 control individuals. 

The study was conducted in accordance with the principles of the Declaration o/f Helsinki and good clinical practices. Written informed consent of the patients and of parents was obtained before conducting the genetic analyses.

## RESULTS

Homozygous ROR2 mutations were identified in all patients. Patient 1 had a homozygous mutation c.545G>A (p.C182Y) in exon 5. Bioinformatics analysis revealed the mutation to be pathogenic; SIFT score, PPH2 score and MT score were 0, 0.998 (sensitivity 0.18, specificity 0.98) and 194 respectively. Patient 2 and his elder sib had a homozygous missense mutation c.227G>A or p.G76D in exon 3. Bioinformatics analysis revealed the mutation to be pathogenic; SIFT score, PPH2 score and MT score were 0, 1.0 (sensitivity 0.00, specificity 1.00) and 94 respectively. Patient 3 had a homozygous mutation in exon 6 viz. c.668G>A or p.C223Y. Bioinformatics analysis revealed the mutation to be pathogenic; SIFT score, PPH2 score and MT score were 0, 1.0 (sensitivity 0, specificity 1) and 194 respectively these mutations were confirmed in the heterozygous state in the respective parents. They were not present in 100 normal individuals. Please see Figure 1 for sequence chromatograms. 

Fetal mutation analysis performed on DNA extracted from the chorionic villus sample of the mother of patient 1 showed absence of mutation p.C182Y. The pregnancy led to the birth of a normal healthy baby. 

## DISCUSSION

RS was considered in the patients presented above who all presented with acromesomelic dwarfism, characteristic facial and genital abnormalities along with the radiological findings of mesomelia and costo-vertebral segmentation defects. In 1969, Robinow et al (1) described a new dwarfing syndrome with mesomelic limb shortening, hemivertebrae and genital hypoplasia (1). Robinow (8) proposed the term “fetal facies” to describe the characteristic facial appearance.

Over 100 cases of RS that cover most ethnic groups have been reported to date. The incidence is reported to be 1:500 000, with equal frequency in males and females. The prevalence is low, as 5%-10% of the children die in infancy because of cardiac problems. Clusters of cases with the autosomal recessive form have been reported from Turkey, Oman, Czechoslovakia, Brazil and Pakistan (9). Only two cases have been published from India. Singh et al (10) reported a case from Varanasi. Kulkarni and Reddy (11) in 2004 reported an 8 years old girl born of consanguineous parentage from Karnataka. Mutation testing was not performed in these two cases.

The characteristic facial features of the syndrome include a large head, a prominent forehead, facial nevus (23%), a flat nasal bridge, a short upturned nose, mandibular hypoplasia, ocular hypertelorism, S- shaped lower eyelids, a triangular mouth with downturned angles and micrognathia. The oral abnormalities include tented upper lip, exposed incisors, gum hypertrophy, dental misalignment, crowded teeth, delayed loss of deciduous teeth, retained molar teeth, notching of teeth, macroglossia, absent or rudimentary uvula (18%), cleft lip and cleft palate (9%) (12). All our cases had the typical craniofacial features of RRS.

The skeletal features include mesomelic shortening of limbs with disproportionate shortening of forearms, short stature with growth delay, scoliosis (50%), hemivertebrae, absent, fused, split ribs, fused carpal bones, brachydactyly (89%) and feet and nail hypoplasia. Genital abnormalities include micropenis, cryptorchidism, primary hypogonadism, vaginal atresia, hypoplasia of labia majora and ambiguous genitalia (13).

Growth hormone deficiency and partial insensitivity of Leydig cells to HCG, low basal testosterone in prepubertal boys and a defective sex-steroid feedback mechanism are also reported (14,15). A low normal response of Leydig cells to HCG was also shown to be present in our first patient.

All patients with the recessive form of RS suffer from vertebral segmentation abnormalities, resulting in kyphoscoliosis and chest deformities. Thoracic vertebrae are commonly fused with frequent hemivertebrae; hence, this anomaly was previously known as cost vertebral segmentation defect with mesomelia syndrome (16). Ribs are also commonly deformed. Hemivertebrae and scoliosis are present in more than 75% of patients with the recessive form, but in less than 25% of patients with the dominant form. Mazzeu et al (4) clinically categorized the patients into recessive and dominant forms based on the presence of rib fusions. Patton and Afzal (9) showed that patients with RRS have a height in the range of -2 SD or less, whereas AD cases have milder phenotype and could have height within normal ranges. The AD cases have only mild shortening of the forearms as compared to marked shortening in AR cases. AR cases have radial head dislocation and tented upper lip which are absent in AD cases. AR cases have 10% mortality rate whereas AD cases do not have increased mortality (9).

Congenital heart defects are frequent in RRS. Fifteen percent of cases have bicuspid aortic valve (seen in our patients), tetralogy of Fallot, atrial septal defect, ventricular septal defect, coarctation of aorta, but the commonest appears to be pulmonary stenosis (9).

Carriers of RRS show normal phenotype. In our family 2, the mother had mild physical dysmorphism with short stature. This finding has not been previously reported in heterozygous carriers of RRS. 

Autosomal dominant RS, spondylothoracic dysostosis (STD), Aarskog syndrome, I-cell disease, brachydactyly with dysmorphic features, omodysplasia were considered in the differential diagnosis. AD RS is similar but less severe than the autosomal recessive (ROR2-related) form, especially regarding the skeletal defects (4). Missense mutations in WNT5A that result in amino acid substitutions of highly conserved cysteines have been reported in AD RS (17). ROR2 has recently been identified as a putative WNT5A receptor. STD (known as Jarcho-Levin syndrome caused due to mutations in MESP2 gene is characterized by a short and rigid neck, short thorax, protuberant abdomen and inguinal and umbilical hernias, severe shortening of the spine with fusion of the ribs posteriorly at the costo-vertebral junctions. Presence of mesomelia and genital ambiguity as well as absence of rigid neck helped us to clinically exclude the above diagnosis in our patients. Dysostosis multiplex, long bone deformities and contractures seen in I-cell disease are not seen in RS. In cases of Aarskog syndrome, facial features are similar with wide-spaced eyes, anteverted nostrils and long philtrum. The mouth may be wide; however the upper lip is not tented. Stature is between the 3rd-10th centiles and rhizomelia may be present. Vertebral abnormalities are not observed. The shawl scrotum and lax ligaments of Aarskog syndrome are not found in ROR2-related RS. Omodysplasia is similar to ROR2-related RS, with short limbs and radial dislocation; however, no genital abnormalities are present (18).

ROR2 contains nine exons and encodes a 4092 bp transcript. The protein product consists of 943 amino acids and is an orphan receptor tyrosine kinase that binds to an unidentified ligand. ROR2 -related RS is caused by missense, nonsense, or frameshift mutations distributed throughout the gene, affecting the regions encoding both the extracellular and the intracellular domains of the protein, although homozygous or compound heterozygous mutations encoded by exons 3, 5, 7, 8, are commonly reported (19). Van Bokhoven et al (2) reported mutations in seven of 11 families (by analysis of exons 2-9 only) and Afzal et al (3) reported mutations in ten of ten families investigated. Although, exonic deletions have been reported in the literature the frequency of deletions and duplications in this disorder is not known (20). The Human Gene Mutation Database lists 26 mutations in ROR2 gene with 17 mutations correlating with Robinow AR phenotype, nine correlating with Brachydactyly type B phenotype (http://www.hgmd.cf.ac.uk/ac/gene.php?gene=ROR2) (21). The orphan receptor tyrosine kinase protein contains distinct motifs including an immunoglobulin like (Ig) domain (amino acids (aa) 55 -145), a frizzled-like cysteine rich domain (aa 169-303) and a kringle domain (aa 316 to 394) in the extracellular region, a transmembrane section (aa 404-424) and an intracellular region with tyrosine kinase (aa 473-746), serine/threonine rich (aa 753-782 and aa 859-882) and proline rich structures (aa 784-857) (http://www.uniprot.org/uniprot/Q01974) (22). Various mutations are described in the frizzled domain are p.C182Y (2), p.R184C and p.R189W (3), p.Y192D (23), p.R205X (3). The p.C182Y mutation seen in our patient was previously described in a Turkish patient with RRS (2). Ali et al (23) and Chen et al (24) have shown that mutations in the frizzled domain lead to loss of function due to retention of the mutated ROR2 protein inside the endoplasmic reticulum (ER) due to misfolding. The protein is then degraded using ER associated protein degradation and does not reach the plasma membrane. All the above patients have severe phenotype like our cases 1 and 3. The mutation p.G76D seen in our patient occurs in the Ig like domain. Previously, another mutation R119X in the Ig like domain has been reported by Tufan et al (19) in a patient with RRS. However the phenotype in the Turkish case reported by Tufan et al (19) is severe, whereas our cases (family 2) are mild because they lack the rib and vertebral findings of RRS.

In conclusion, this paper reports the findings on three families with RRS which were identified in India. Three mutations in ROR2 gene, two novels and one previously known mutation were observed in these patients. One patient with mild mutation was identified. Knowledge about the molecular basis of the disorder enabled us to make a prenatal diagnosis and to provide accurate genetic counseling to the families.

**Acknowledgement**

The authors acknowledge the help of Mrs Pratima Kondurkar, Mrs Shiny Babu, Mrs Aruna D’Souza and Mrs Rashmi Adhia for their technical help. The authors thank the families of the affected patients for their participation.
